# A Unique Combination of Nutritionally Active Ingredients Can Prevent Several Key Processes Associated with Atherosclerosis *In Vitro*

**DOI:** 10.1371/journal.pone.0151057

**Published:** 2016-03-07

**Authors:** Joe W. E. Moss, Thomas S. Davies, Iveta Garaiova, Sue F. Plummer, Daryn R. Michael, Dipak P. Ramji

**Affiliations:** 1 School of Biosciences, Cardiff University, Cardiff, United Kingdom; 2 Cultech Limited, Baglan Industrial Park, Port Talbot, United Kingdom; Universitat de Lleida-IRBLLEIDA, SPAIN

## Abstract

**Introduction:**

Atherosclerosis is the underlying cause of cardiovascular disease that leads to more global mortalities each year than any other ailment. Consumption of active food ingredients such as phytosterols, omega-3 polyunsaturated fatty acids and flavanols are known to impart beneficial effects on cardiovascular disease although the combined actions of such agents in atherosclerosis is poorly understood. The aim of this study was to screen a nutritional supplement containing each of these active components for its anti-atherosclerotic effect on macrophages *in vitro*.

**Results:**

The supplement attenuated the expression of intercellular adhesion molecule-1 and macrophage chemoattractant protein-1 in human and murine macrophages at physiologically relevant doses. The migratory capacity of human monocytes was also hindered, possibly mediated by eicosapentaenoic acid and catechin, while the ability of foam cells to efflux cholesterol was improved. The polarisation of murine macrophages towards a pro-inflammatory phenotype was also attenuated by the supplement.

**Conclusion:**

The formulation was able to hinder multiple key steps of atherosclerosis development *in vitro* by inhibiting monocyte recruitment, foam cell formation and macrophage polarisation towards an inflammatory phenotype. This is the first time a combination these ingredients has been shown to elicit such effects and supports its further study in preclinical *in vivo* models.

## Introduction

Cardiovascular disease (CVD) related events, such as myocardial infarction (MI) and stroke, are the leading causes of global death every year. The World Health Organisation estimated that there were approximately 17.5 million deaths from CVD-related events in 2012 [1] and this figure has been predicted to rise to 23.3 million by 2030 due to the global increase in obesity and diabetes, and the incorporation of a westernised lifestyle in developing countries [1]. Atherosclerosis is the major underlying cause of CVD, emphasising the need to develop novel approaches to support disease prevention.

Atherosclerosis is a chronic, inflammatory disease of the vasculature characterised by the formation of lipid laden foam cells [2–5]. The initial trigger of atherosclerosis is the accumulation and trapping of ApoB-containing lipoproteins, such as low-density lipoprotein (LDL), in the intima of medium and large arteries [3–5]. Modification of the trapped LDL, particularly oxidised (ox)-LDL, instigates an inflammatory response in the nearby endothelial cells (ECs) [3–5]. Activated ECs then release pro-inflammatory cytokines and chemokines, such as macrophage chemoattractant protein-1 (MCP-1), which direct circulating monocytes and T lymphocytes to the site of oxLDL accumulation [3–5]. Additionally, the ECs also begin to express cell adhesion molecules on their surface, including intercellular adhesion molecule-1 (ICAM-1) and vascular cell adhesion molecule-1 (VCAM-1), as well as P- and E-selectins, which aid the adhesion of circulating monocytes to the site of oxLDL accumulation [3–5]. Once in the intima, the monocytes differentiate into macrophages which are then able to uptake oxLDL and develop into foam cells [3–5]. Over time, the continued and aberrant recruitment of macrophages to the site of insult results in the accumulation of foam cells that eventually develop into an unstable atherosclerotic plaque that, upon rupture, leads to thrombosis, MI and stroke [3–5].

Numerous macrophage phenotypes reside within atherosclerotic lesions and this population is largely composed of classical M1 or alternative M2 polarised macrophages [3, 5]. Under normal physiological conditions, M2 polarisation occurs as a result of IL-4 stimulation and is usually associated with the resolution of inflammation and repair whereas M1 macrophages are activated by stimuli such as interferon-γ (IFN-γ), lipopolysaccharide (LPS) and oxLDL and participate in pro-inflammatory actions towards bacterial infections during the innate immune response [6]. However, due to their pro-inflammatory nature it is thought that the presence of M1 macrophages in atherosclerotic plaques can drive disease progression [7, 8] and it has been shown that this macrophage subset often localises to rupture-prone shoulder areas of atherosclerotic plaques [8]. Approaches to reduce M1 accumulation may result in more stable atherosclerotic plaques and also represent an ideal target for disease intervention.

It has been estimated that approximately 70% of cardiovascular disease related events are not prevented by the current therapies which includes the use of statins [9]. These are the most extensively used class of cholesterol lowering therapy for CVD and their primary function is to inhibit the enzyme 3-hydroxy-3-methylglutaryl-CoA, which catalyses the rate-limiting step in cholesterol biosynthesis [4]. However, a significant minority of people receiving statins are unable to lower their LDL levels even at the maximal dose [4] and are prone to adverse side effects such as increased risk of type 2 diabetes [10, 11] stressing the need to develop novel approaches to prevent atherosclerosis. It has been known for some time that the consumption of various active food ingredients can improve cardiovascular health. For example, numerous epidemiological studies [12, 13] and clinical trials [14, 15] examining the effects of fish oil consumption, particularly the omega-3 polyunsaturated fatty acids (ω-3 PUFAs) eicosapentaenoic acid (EPA) and docosahexaenoic acid (DHA), have reported lower plasma cholesterol levels and a reduced risk of a MI. EPA and DHA have also been shown to dampen the inflammatory response by macrophages by directly binding to G protein-coupled receptor (GP)-120 present on the cell surface [16] which is likely to contribute to the observed anti-foam cell effects of these fatty acids [17]. Flavanoids, a group of secondary plant metabolites that can be found in foods such as cocoa beans and specific flavonoid subgroups, such as flavanols (catechins), have also been shown to reduce the expression of pro-inflammatory genes [18], and circulating LDL levels *in vivo* [19]. An abundant form of catechin, epigallocatechin-3-gallate, has been shown to impart an anti-inflammatory effect on macrophages by directly binding to the cell surface laminin receptor (67LR) [20] and by regulating the expression of a range of trancription factors including nuclear factor κB (NF-κB) (reviewed extensively in [21]). Phytosterols (PS) are cholesterol homologues found in plants that have been shown to reduce plasma LDL levels in both observational and clinical studies [22, 23] by competitive exclusion of cholesterol from micellar space in the intestinal lumen and modulation of enterocyte cholesterol trafficking [24]. PS also have a direct effect on cholesterol homeostasis in macrophage-derived foam cells by regulating the expression of key cholesterol transport genes such as ATP-binding cassette transporter (ABC)A-1 and ABCG-1 [25].

The objective of this study is to assess the ability of a dual-action nutritional supplement comprised of mixed PS, ω-3 PUFA-rich fish oil and flavanol rich cocoa extract in a readily absorbable emulsified format [26, 27] to prevent the development of atherosclerosis by, firstly, reducing the amount of cholesterol absorbed in the intestines by the action of PS, followed by inhibition of the cellular mechanisms driving the disease after absorption. The supplement has been designed to present physiologically relevant doses of the active ingredients in a series of established *in vitro* models of atherosclerosis in order to demonstrate efficacy.

## Methods

### Regents and cell culture

All reagents were purchased from Life Technologies (Paisley, UK), unless otherwise stated. EPA, DHA, campesterol and β-sitosterol were purchased from Cambridge Biosciences (Cambridge, UK). Stigmasterol and (+)-catechin were purchased from Sigma-Aldrich (Poole, UK). Human acute monocytic leukemia cell line (THP-1; obtained directly from ECACC, Salisbury, UK (Catalogue number: 88081201)), primary human monocyte derived macrophages (HMDM) and Raw264.7 murine macrophage (obtained directly from Sigma-Aldrich, Poole, UK (Catalogue number: 85062803-VL)) were grown in complete RPMI-1640 (Lonza, Manchester, UK) supplemented with 10% (v/v) heat-inactivated fetal calf serum (FCS), penicillin (100 U/ml), streptomycin (100 μg/ml) and L-glutamine (2 mmol/l) at 37°C in a humidified atmosphere containing 5% (v/v) CO_2_. THP-1 monocytes were differentiated into macrophages by incubation with 160 nM phorbol-12-myristate-13-acetate (PMA; Sigma-Aldrich, Poole, UK) for 24 hours to ensure high expression levels of genes implicated in the control of macrophage foam cell formation [28, 29]. HMDM were isolated from buffy coats supplied by the Welsh Blood service using Ficoll-Hypaque purification described elsewhere [29]. Ethical approval and informed consent for each donor was granted by the Welsh Blood Service for the use of human blood samples.

### Formulation dosage and application to cells

The nutritional supplement formulation (S1 Table) was provided by Cultech Limited (Port Talbot, UK) as a concentrated stock (500x) designed to deliver each active ingredient at doses approximating to reported *in vivo* serum levels with a 1x dose composed of 180 μg/ml fish oil (delivering 30 μg/ml EPA, 19.7 μg/ml DHA [26]), 56 μg/ml of mixed PS (delivering 10 μg/ml Stigmasterol [30], 13.9 μg/ml Campsterol, 27.2 μg/ml Sitosterol and 1.672 μg/ml Brassicasterol) and 7.25 μg/ml cocoa extract (delivering 5 μmol/ml catechins [31]). A PS-free version of the formulation was also provided. Fatty acid free bovine serum albumin (BSA; 100 μg/ml per 1x dose) was included in both formulations to act as a physiologically relevant protein carrier and to facilitate the emulsification of the formulations and allow their natural dispersion in an aqueous medium. BSA alone was used as the vehicle control at an appropriate concentration and the formulation was applied to the cells diluted in complete culture media unless otherwise stated. EPA, DHA, (+)-catechin, stigmasterol, campsterol and β-sitosterol were applied to the cells (at doses equivalent to those present in the complete formulation (x1)) in dimethyl sulfoxide (DMSO) that was also included at the relevant concentration as the vehicle control for these experiments.

### Cell viability and proliferation assays

The cell supernatants from THP-1 macrophages (4.11x10^5^ cells/cm^2^) were removed and assayed for lactate dehydrogenase (LDH) content using the Pierce LDH cytotoxicity assay (Thermo Scientific, Waltham, USA) in accordance with the manufacturer’s instructions. The remaining cells were then stained with 0.2% (w/v) crystal violet solution (in 10% (v/v) ethanol) for 5 minutes at room temperature before washing the cells three times in warm PBS (pH 7.4). Intracellular crystal violet was then solubilised in 0.1 M NaH_2_PO_4_ (in 50% (v/v) ethanol) and the absorbance was read at 570 nm using a colorimetric spectrophotometer. Viability (LDH assay) and proliferation (crystal violet) were expressed as a percentage of the BSA vehicle control that was arbitrarily assigned as 100%.

### RNA extraction, reverse transcription, and quantitative PCR

RNA was extracted from THP-1 macrophages (1.28x10^5^ cells/cm^2^), HMDM (1.28x10^5^ cells/cm^2^) or Raw264.7 macrophages (5.13x10^4^ cells/cm^2^) using RiboZol^™^ RNA extraction reagent (Amresco, Solon, US) in accordance with the manufacturer’s instructions and total RNA concentrations were quantified using a NanoDrop 2000 spectrophotometer (Thermo Scientific, Waltham, USA). cDNA was then reverse transcribed from 1000 ng of RNA as previously described [32]. Quantitative polymerase chain reaction (qPCR) was performed on 10 ng cDNA using SYBR Green JumpStart^™^ Taq Readymix^™^ and 50 nM of each oligonucleotide primer was used (see S2 Table). Fold changes in gene expression were calculated using 2^−(ΔCt1−ΔCt2)^, where ΔCt represents the difference between the threshold cycle (Ct) for each target gene and Glyceraldehyde 3-phosphate dehydrogenase (GAPDH) transcript levels for human studies or β-actin mRNA transcript levels for murine studies. Initial melting (94°C for 120 seconds) was followed by 40 cycles of melting (95°C for 30 seconds), annealing (60°C for 60 seconds (66°C for Inducible Nitrogen Oxide Synthase (iNOS) and Arginase-2 (Arg2)), and extension (72°C for 60 seconds)). The inclusion of reverse transcriptase negative controls and melting curve analysis in each experiment confirmed there was no amplification from genomic DNA. The amplicon size of all primers sets were confirmed by agarose gel electrophoresis (data not shown).

### Monocyte Migration

Monocyte migration was assessed by adding undifferentiated THP-1 monocytes (alone or with formulation or with individual bioactive components; 1x10^6^ cells/ml) to the apical compartment of Falcon^®^ cell culture inserts (8 μm pore size) that were housed in Falcon^®^ 12-well companion plates (VWR Jencons, Lutterworth, UK) containing complete RPMI media supplemented with MCP-1 (20 ng/ml; Peprotech, London, UK). Following incubation, apical medium was aspirated and the underside of the porous membrane washed into the wells to capture all migrating cells. Monocyte numbers present in the basolateral (lower) compartment were counted using a haemocytometer and monocyte migration is expressed as a fold-change compared to the proportion of cells that moved from the apical compartment into the basolateral compartment in response to MCP-1 alone and has been arbitrarily set as 1.0.

### Cholesterol efflux assay

Cholesterol efflux assays were carried out following a previously described protocol with minor adaptations [32]. Briefly, THP-1 macrophages (1.28x10^5^ cells/cm^2^) were incubated with acetylated (Ac)LDL (25 mg/ml; Biotrend, Cologne, Germany) and [4-^14^C]cholesterol (0.5 mCi/ml; Amersham, Buckinghamshire, UK) in media containing 0.2% (v/v) fatty acid free BSA for 24 hours before they were treated for a further 24 hours with the previously described media containing Apolipoprotein A-I (ApoA-I) (10 mg/ml; Sigma-Aldrich, Poole, UK) with or without the formulation. Cell supernatants were then collected and the remaining cells were solubilised in 0.2 M NaOH. Cholesterol efflux was calculated as the percentage radioactivity in the supernatant versus total radioactivity (cells and supernatant) as measured by a liquid scintillation counter. Cholesterol efflux was expressed as a fold change of the vehicle control that was arbitrarily assigned as 1.0.

### Data analysis

The normality of all data sets was tested using Shapiro-Wilk test for normality and confirmed with histograms and Q-Q plots. Any required data transformations are stated in the figure legends. *P* values were determined using one-way ANOVA with Dunnett T3 (unequal variance) or Dunnett (equal variance) post-hoc analysis and significance was defined when *p*<0.05. All statistics were performed using SPSS statistical software package version 20.0. Data are presented as means (± standard error of the mean (SEM)).

## Results

### The formulation does not impart a cytotoxic effect on human macrophages *in vitro*

**Fig 1:**
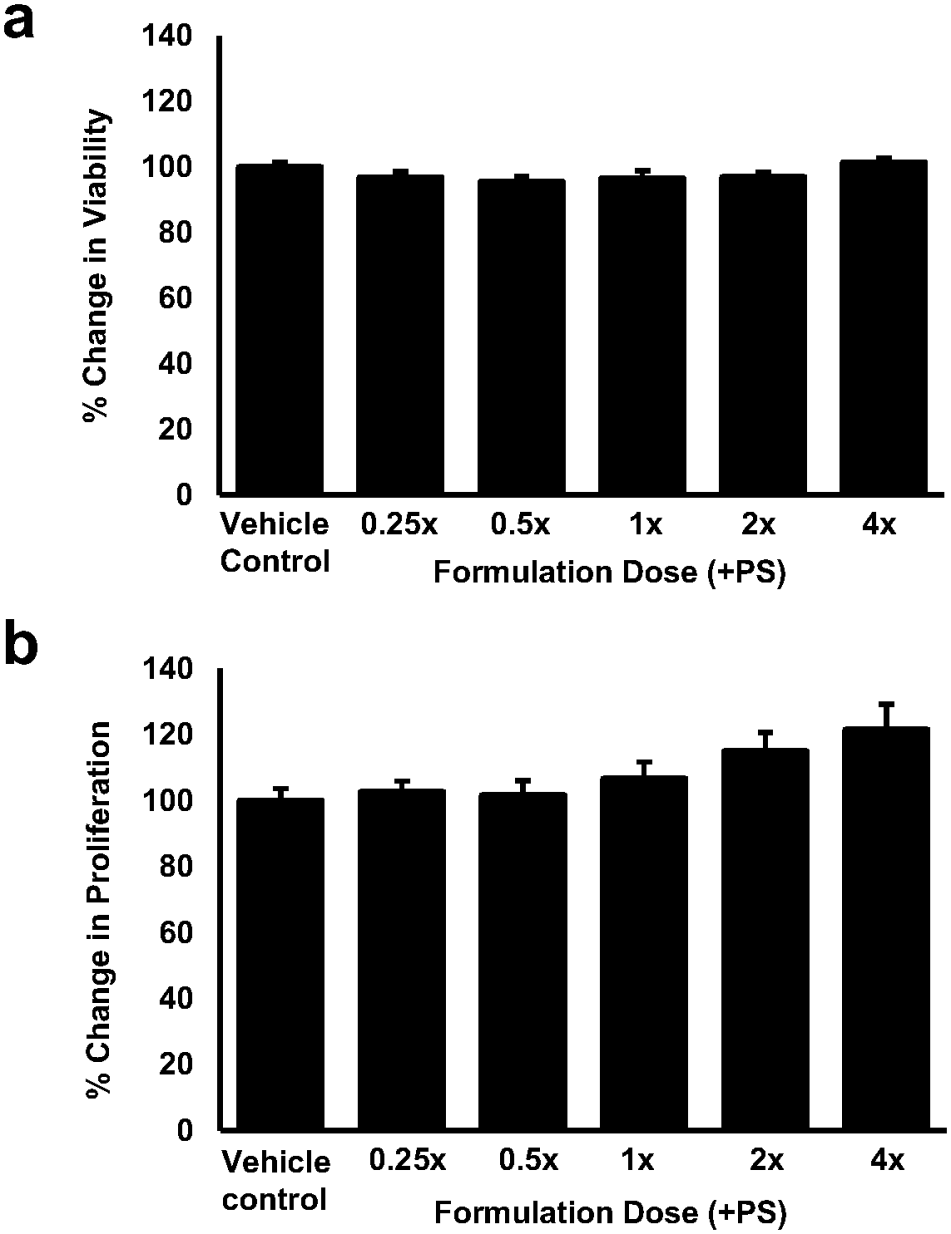
Physiologically relevant doses of the formulation have no detrimental effect on human macrophage viability or proliferation. Cell viability (a) or proliferation (b) was assessed in PMA differentiated THP-1 macrophages that were treated with vehicle (vehicle control) or various doses of the complete formulation (+PS) for 24 hours. Data were normalised to the vehicle control that has been arbitrarily assigned as 100%. The data are presented as the mean ± SEM from three independent experiments. Statistical analysis was performed using one-way ANOVA with Dunnett T3 post-hoc analysis.

The relative health and activity of the human THP-1 monocyte-derived macrophages in the presence of the formulation was confirmed by two independent assays. As shown in Fig 1, no significant differences in cell viability (Fig 1a: LDH assay) or cell proliferation/activity (Fig 1b: crystal violet assay) were observed in response to 24 hours incubation with the complete formulation (+PS) at a range of doses when compared to the vehicle control.

### The formulation inhibits the IFN-γ induced expression of MCP-1 and ICAM-1 mRNA transcripts in macrophages

**Fig 2:**
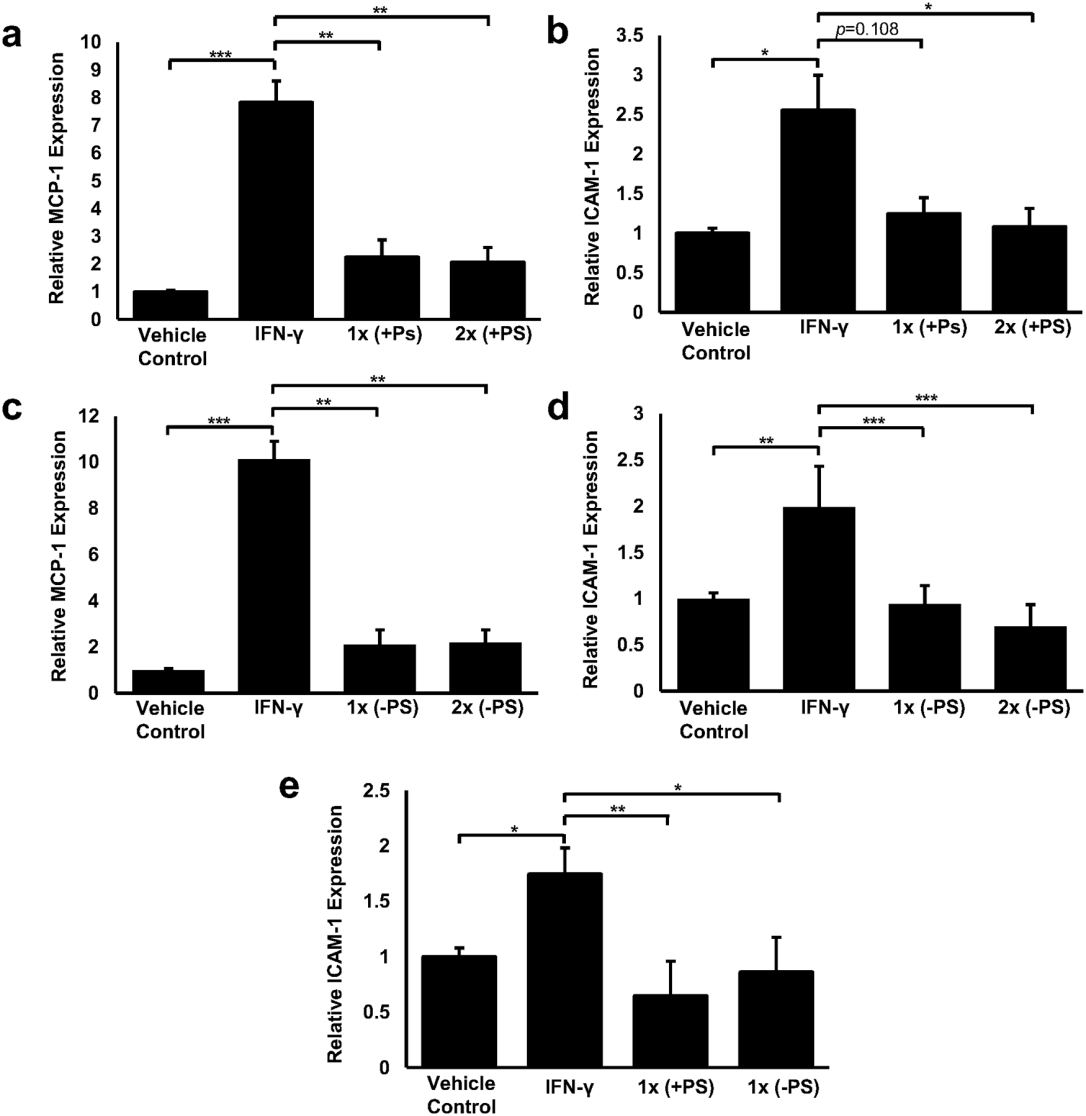
Physiologically relevant doses of the formulation can inhibit the IFN-γ induced expression of MCP-1 and ICAM-1 in human macrophages. Gene transcript levels of MCP-1 (a and c) and ICAM-1 (b, d and e) were assessed in PMA differentiated THP-1 macrophages (a, b, c and d) or HMDM (e) that were either treated with vehicle (vehicle control) or with IFN-γ (250 U/ml) or IFN-γ (250 U/ml) in the presence of the complete formulation (+PS; a, b and e) or with IFN-γ (250 U/ml) in the presence of the formulation lacking PS (-PS; c, d and e) for 3 hours. Gene transcript levels were calculated using the comparative Ct method and normalised to GAPDH levels with values from vehicle treated cells given an arbitrary value of 1. The data are presented as the mean ± SEM from three (a, b, c and d) or four (e) independent experiments. Statistical analysis was performed using a one-way ANOVA with Dunnett T3 post-hoc analysis on log-transformed data where * *p* <0.05, ** *p* <0.01 and *** *p* <0.001.

MCP-1 and ICAM-1 are both considered to be robust markers of inflammation that are associated with atherosclerosis [5] and their expression is known to be induced by IFN-γ [33]. To this end, IFN-γ stimulation significantly increased the transcript levels of MCP-1 (Fig 2a; 7.83-fold increase (*p*<0.001), Fig 2c; 10.13-fold increase (*p*<0.001)) and ICAM-1 (Fig 2b; 2.55-fold increase (*p* = 0.014), Fig 2d; 1.99-fold increase (*p* = 0.001)) in human THP-1 monocyte-derived macrophages when compared to the vehicle control. However, in the presence of 1x and 2x doses of the complete formulation, significant 71.3% (*p* = 0.004) and 73.6% reductions (*p* = 0.001), respectively, in IFN-γ induced MCP-1 transcript levels were observed when compared to cells treated with IFN-γ alone (Fig 2a). Likewise, 51.1% (*p* = 0.108) and 57.5% (*p* = 0.010) reductions were observed in IFN-γ induced ICAM-1 expression in response to the same doses, although the 1x dose failed to reach significance (Fig 2b). As seen is Fig 2c, comparable effects were also apparent in response to a formulation that lacked the PS component where the 1x and 2x doses were able to significantly reduce IFN-γ induced MCP-1 transcript levels by 79.1% (*p* = 0.001) and 78.2% (*p* = 0.002), respectively, when compared to the IFN-γ treated cells. IFN-γ induced ICAM-1 expression was also significantly reduced in the presence of 1x and 2x doses lacking PS (by 52.8% (*p*<0.001) and 64.7% (*p*<0.001) respectively) when compared to the IFN-γ treated cells (Fig 2d). These effects were partly confirmed in primary HMDM where IFN-γ induced expression of ICAM-1 (1.75-fold increase (*p* = 0.016)) was significantly decreased the presence of the complete formulation and that lacking PS by 63.9% (*p* = 0.002) and 50.6% (*p* = 0.013) respectively (Fig 2e). No significant differences were observed between the complete formulation and that lacking PS for either gene in THP-1 or HMDM. In addition, the observed changes in MCP-1 and ICAM-1 gene expression also appear to be conserved in murine Raw264.7 macrophages although slight differences in dosing are apparent (S1 Fig).

### The formulation inhibits the migration of human monocytes towards MCP-1

**Fig 3:**
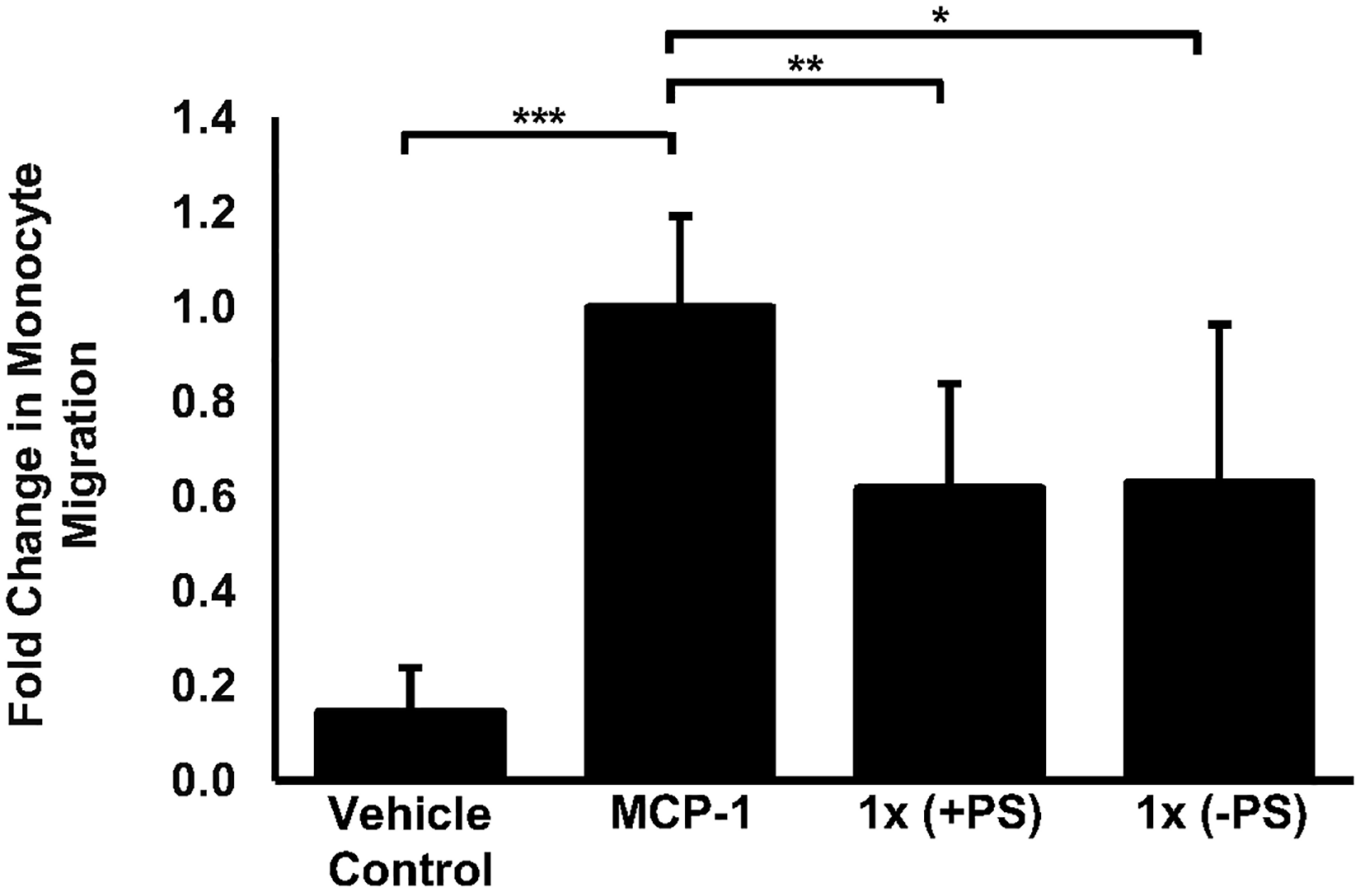
A physiologically relevant dose of the formulation can inhibit MCP-1 induced migration of human monocytes. Cellular migration was assessed using THP-1 monocytes that were treated with vehicle (vehicle control) or treated with MCP-1 (20 ng/ml) alone or with MCP-1 (20 ng/ml) in the presence the complete formulation (+PS) or with MCP-1 (20ng/ml) in the presence the formulation lacking PS (-PS) for 3 hours. Monocyte migration is expressed as a fold-change compared to the proportion of cells that moved from the apical compartment into the basolateral compartment in response to MCP-1 alone that has been arbitrarily set as 1. The data are presented as the mean ± SEM from four independent experiments. Statistical analysis was performed using a one-way ANOVA with Dunnett T3 post-hoc analysis where ** *p* <0.01 and *** *p* <0.001.

The recruitment of monocytes to the activated endothelium in response to chemokines such as MCP-1 is a critical early step in the development of atherosclerosis [5] and these actions were confirmed in our system as a significant 6.84-fold induction (*p*<0.001) in monocyte recruitment in cells treated with MCP-1 alone was observed when compared to the vehicle control (Fig 3). Upon the inclusion of the complete formulation (1x), monocyte recruitment in response to MCP-1 was significantly reduced (38.2%, *p* = 0.001) when compared to cells treated with MCP-1 alone. Similarly, incubation with the formulation lacking PS resulted in a 37% (*p* = 0.023) reduction in migration when compared to MCP-1 alone treated cells. No significant difference in monocyte migration was observed between the complete formulation and that lacking PS.

### The formulation improves the efflux of cholesterol from human macrophages

**Fig 4:**
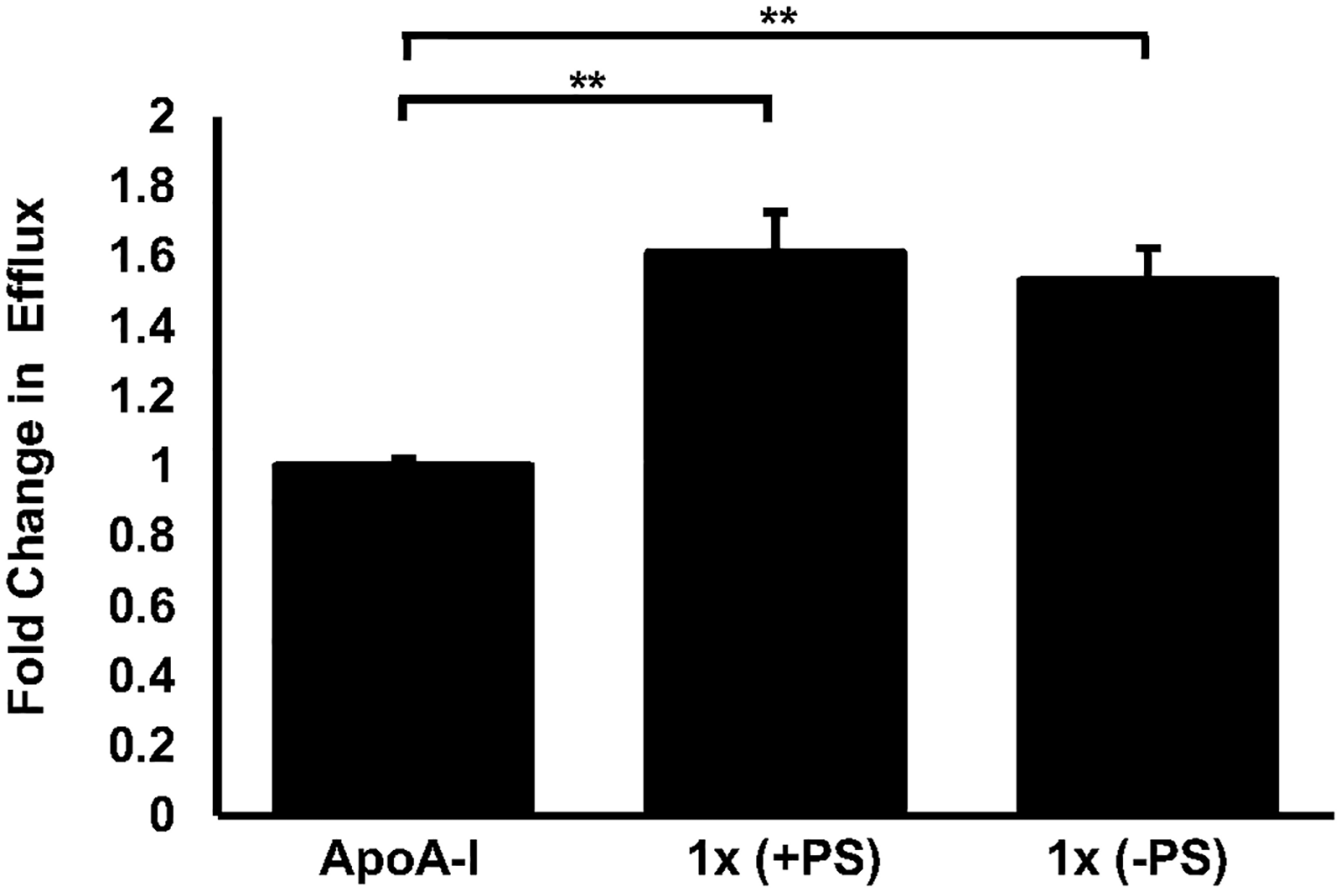
A physiologically relevant dose of the formulation can induce cholesterol efflux from human macrophage foam cells. Cholesterol efflux was assessed in 24 hour [4-^14^C]cholesterol loaded PMA-differentiated THP-1 cells in the presence of AcLDL that were treated with ApoA-I (10 μg/ml) in the presence of vehicle or ApoA-I in the presence of the complete formulation (+PS) or ApoA-I in the presence of the formulation lacking PS (-PS) for 24 hours. Data were normalised to the ApoA-I, vehicle treated sample that has been arbitrarily assigned as 1. The data are presented as the mean ± SEM from three independent experiments. Statistical analysis was performed using a one-way ANOVA with Dunnett T3 post-hoc analysis where ** *p* <0.01.

After 24 hours stimulation with ApoA-I, human THP-1 macrophage-derived foam cells were able to efflux approximately 20.2% of their intracellular radiolabelled cholesterol content (Fig 4). Significant fold changes in cholesterol efflux were observed in cells which were either co-incubated with the complete formulation (1x) or the formulation lacking PS (1x) with ApoA-I, 1.61 (*p* = 0.002) and 1.53 (p = 0.001) respectively, when compared to those treated with ApoA-I alone. No significant differences in cholesterol efflux were observed between the complete formulation and that lacking PS.

### The formulation attenuates the expression of M1 phenotype markers in murine macrophages

**Fig 5:**
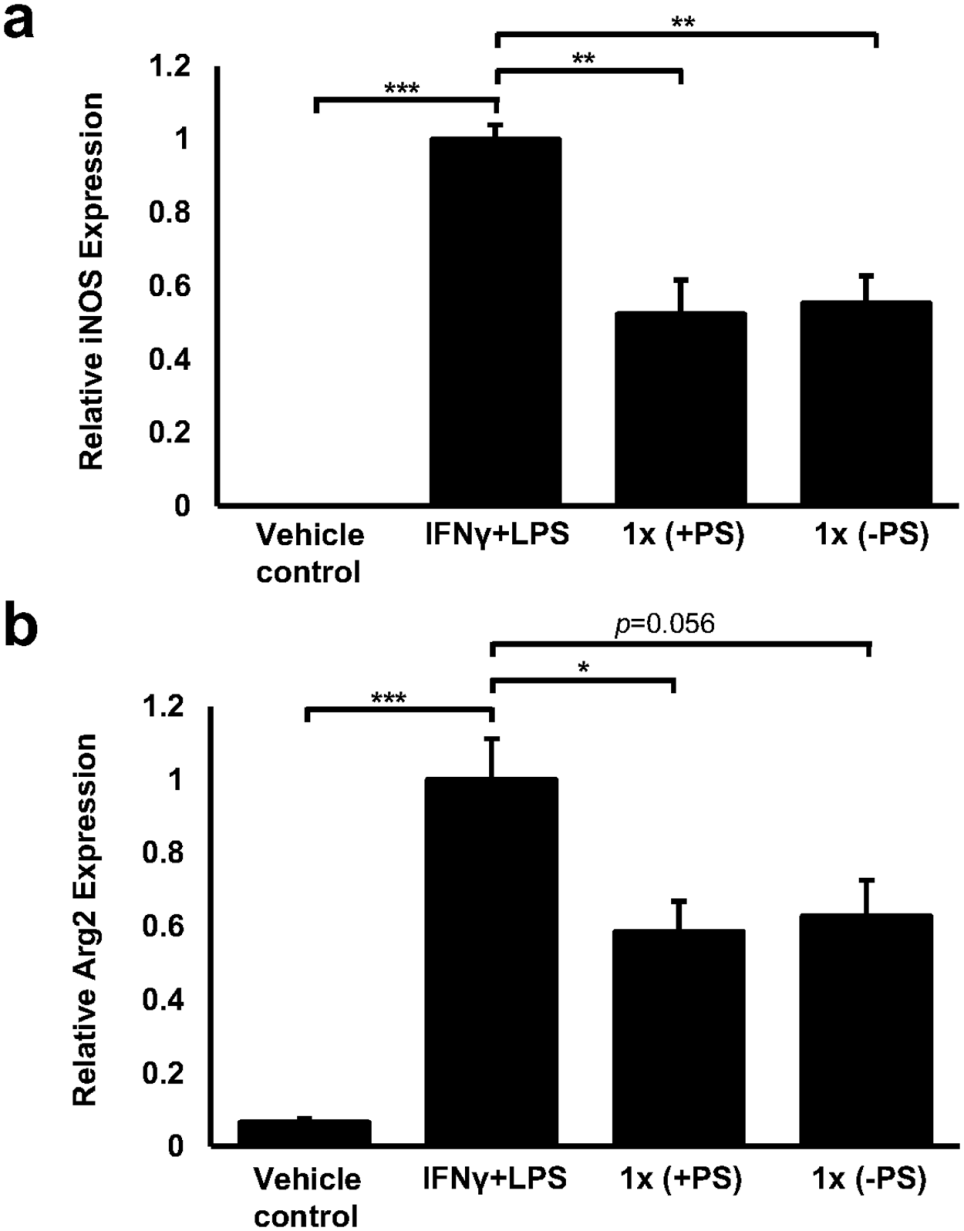
A physiologically relevant dose of the formulation can hinder M1 polarisation in murine macrophages. Gene transcript levels of iNOS (a) and Arg2 (b) were assessed in Raw264.7 murine macrophages that were treated with either vehicle (vehicle control); with IFN-γ (250 U/ml) and LPS (100 ng/ml); with IFN-γ (250 U/ml) and LPS (100 ng/ml) in the presence of the complete formulation (+PS) or with IFN-γ (250 U/ml) and LPS (100 ng/ml) in the presence of the formulation lacking PS (-PS) for 24 hours. Gene transcript levels were calculated using the comparative Ct method and normalised to β-actin levels with the values from IFN-γ and LPS treated cells given an arbitrary value of 1. The data are presented as the mean ± SEM from five independent experiments. Statistical analysis was performed using a one-way ANOVA with Dunnett T3 post-hoc analysis on square root-transformed data where * *p* <0.05, ** *p* <0.01 and *** *p* <0.001.

Co-stimulation of Raw264.7 macrophages with IFN-γ and LPS significantly induced the expression of iNOS (Fig 5a, *p*<0.001) and Arg2 (Fig 5b, *p*<0.001), two robust markers of M1 phenotype [34]. Inclusion of the complete formulation (1x) significantly decreased the expression of both iNOS (Fig 5a) and Arg2 (Fig 5b) by 47.6% (*p* = 0.004) and 41.5% (*p* = 0.015), respectively, when compared to the IFN-γ and LPS treated cells. Likewise, incubation with the formulation lacking PS (1x) significantly reduced the IFN-γ and LPS mediated induction of iNOS (Fig 5a) by 44.6% (*p* = 0.002) whereas a trend towards a 37.2% decrease (*p* = 0.056) was observed for Arg2 (Fig 5b). No significant differences in iNOS or Arg2 expression were observed between the complete formulation and that lacking PS. It should be noted that murine Raw264.7 macrophages were used in these experiments rather than human THP-1 monocyte-derived macrophages in light of evidence suggesting that PMA differentiation promotes an established population of M1 phenotype [35].

### Key bioactive ingredients of the complete formulation can inhibit the migration of human monocytes towards MCP-1

**Fig 6:**
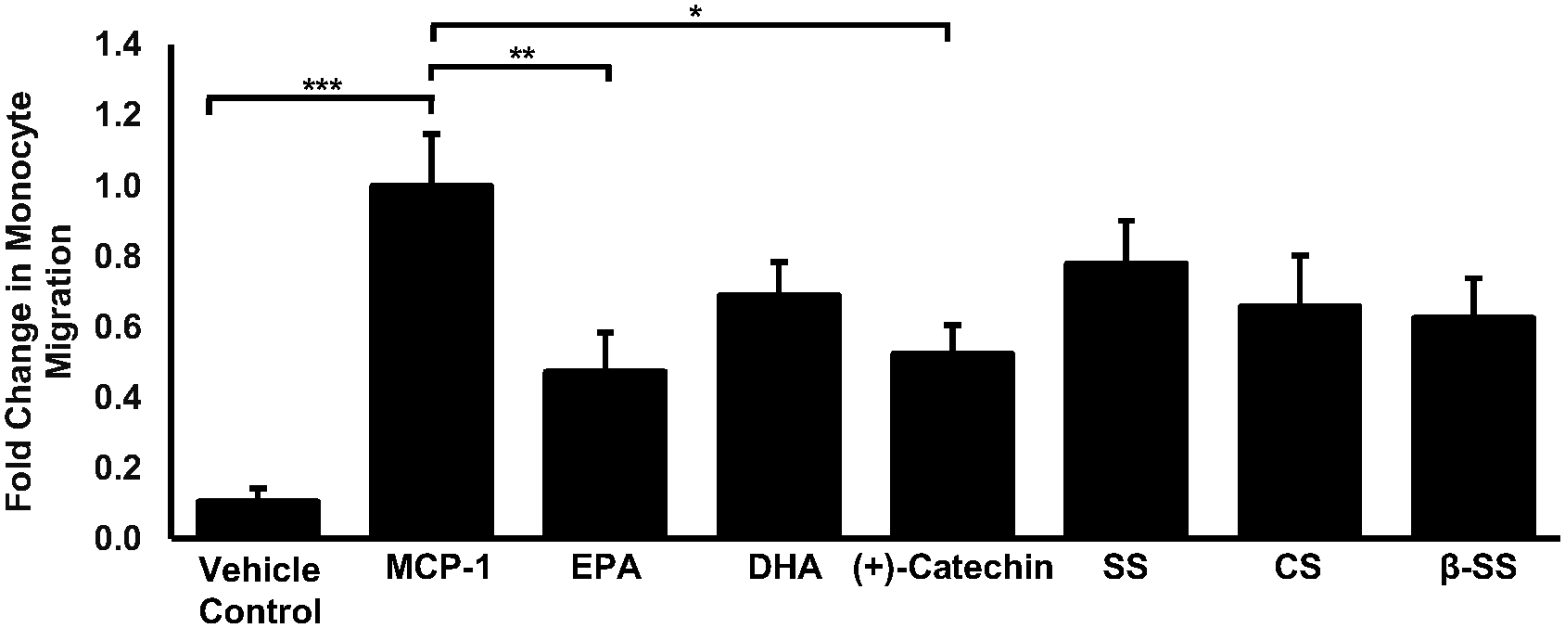
Major bioactive components associated with the complete formulation can inhibit MCP-1 induced migration of human monocytes. Cellular migration was assessed using THP-1 monocytes that were treated with either vehicle (vehicle control) or with MCP-1 (20 ng/ml) or with MCP-1 (20 ng/ml) in the presence of EPA (30 μg/ml), DHA (19.6 μg/ml), (+)-catechin (1.45 μg/ml), stigmasterol (SS, 10 μg/ml), campsterol (CS, 13.9 μg/ml) or β-sitosterol (β-SS, 27.2 μg/ml) for 3 hours. Monocyte migration is expressed as a fold-change compared to the proportion of cells that moved from the apical compartment into the basolateral compartment in response to MCP-1 alone that has been arbitrarily set as 1. The data are presented as the mean ± SEM from four independent experiments. Statistical analysis was performed using a one-way ANOVA with Dunnett post-hoc analysis on Log transformed data where * *p* <0.05, ** *p* <0.01 and *** *p* <0.001.

Similar to that observed in Fig 3, MCP-1 significantly induced (9.61-fold, *p*<0.001) monocyte recruitment when compared to the vehicle control (Fig 6). Significant inhibitions of MCP-1 induced monocyte migration were observed in the presence of EPA (52.7%, *p* = 0.009) or (+)-catechin (47.5%, *p* = 0.02) when compared to cells treated with MCP-1 alone. While they did not reach significance, a trend towards a reduction in monocyte migration was observed in response to DHA, stigmasterol (SS), campsterol (CS) and β-sitosterol (β-SS).

## Discussion

This study shows that a combination of food ingredients can attenuate the expression of two key pro-atherogenic genes, reduce MCP-1 driven monocyte migration, induce cholesterol efflux from macrophage foam cells and retard the polarisation of macrophages towards an M1 (pro-inflammatory) phenotype when assessed using a series of well-established *in vitro* models of atherosclerosis. The ability of physiologically relevant doses of this unique formulation to beneficially modulate multiple key atherosclerotic events highlight the possibility of a nutritionally orientated approach to support the prevention of early disease development.

To date, no other studies have examined the effect of a similar combination of active ingredients on atherosclerosis or key processes associated with this disease although a plethora of *in vivo* studies have demonstrated the anti-atherosclerotic actions of fish oils [36], cocoa extracts [18, 19] and PS [22, 23] in isolation. However, it should be noted that polyphenols, fish oils and PS have all been shown to impart undesirable effects in numerous studies [25, 37–39] although such observations may be the result of the inclusion of single agents in *in vitro* studies that do not consider the potential impact of all other natural food source components, or, in *in vivo* studies, other contributing factors such as the genetic make-up and the presence of various risk factors such as elevated plasma cholesterol levels or a disrupted inflammatory state. In light of this, and due to their ability to directly affect macrophage function [16, 20, 25], the food ingredients used in our study were applied primarily in their natural or most available format, although in some instances individual bioactive components were also used, in a well-established monocyte derived THP-1 macrophage model that shows a highly conserved response with primary HMDM and *in vivo* evidence [29, 32, 40] and, considering the involvement of macrophages at all stages of atherosclerosis [4], in order to facilitate a holistic screening approach.

We first confirmed that no adverse effects on cell viability or proliferation were apparent in response to the application of the food ingredients to human macrophages *in vitro* (Fig 1). These data also ensured that any observed changes in gene expression and/or cell behaviour reported were not as a consequence of altered cell health. In addition, it is worthy of note that PS have been included in the formulation to utilise their ability to reduce the absorption of dietary cholesterol from the intestinal lumen upon regular consumption of the supplement [24]. However, PS are known to cross the intestinal barrier albeit at low levels [41] and individual PS such as stigmasterol and sitosterol, both of which are present in the supplement, have been shown to elicit sometimes opposing atherosclerotic effects on macrophages *in vitro* [25]. Therefore, a formulation lacking PS was assessed in all of our *in vitro* models to determine the potential atherosclerotic effects of systemic PS. To this end, Figs 2, 3, 4, 5 and 6 show that no masked anti-atherosclerotic effect can be attributed to PS and support its inclusion in the formulation.

IFN-γ is a pro-inflammatory cytokine and is considered to be a key regulator of atherosclerosis [3, 5, 42]. It is produced at prominent levels in atherosclerotic lesions by a variety of immune cells including T-lymphocytes, natural killer T-cells and macrophages [43] and is considered a promising therapeutic target due to its key role in all stages of the disease [44]. Studies by our own group have showed that IFN-γ can induce the expression of MCP-1 and ICAM-1 in macrophages that are known to play prominent roles during the recruitment of monocytes to the inflamed endothelium [33]. Here we show that physiologically relevant doses of the formulation, with or without PS, can attenuate the IFN-γ induced ICAM-1 and MCP-1 expression in THP-1 monocyte derived macrophages, physiologically relevant primary HMDM (Fig 2) and murine macrophages (S1 Fig) and may therefore prevent the recruitment of monocytes to the inflamed endothelium—a key stage of early atheroma development. In addition, incubation with either formulation clearly inhibits the movement of monocytes towards MCP-1 (Fig 3) which also alludes to reduced numbers of macrophages at the site of atheroma development and adds functionality to our observed changes in gene expression (Fig 2). Analysis of individual bioactive components contained in the formulation suggest that the effects on monocyte migration maybe mediated by EPA and (+)-catechin (Fig 6), an observation supported by other studies demonstrating reduced monocyte migration/adhesion in response to various catechin isomers [45, 46] and fish oil or ω-3 PUFAs [47, 48]. Reduced serum ICAM-1 levels in response to cocoa extract supplementation [49] and reduced transcript levels of ICAM-1 in EPA stimulated monocytes [50] has also been previously documented. Interestingly, our study also suggests the lack of antagonistic or synergistic effects between the individual formulation components as both EPA and (+)-catechin (Fig 6) appear to attenuate monocyte migration at levels comparable with the complete formulation (Fig 3) though further experiments will be required for firm conclusions.

The formation of macrophage derived foam cells is also considered a major step in atherosclerosis development [4] and in recent years the underlying mechanisms have become a common target for preliminary intervention studies [29, 32]. Here we report that exposure of cholesterol-loaded human macrophages, or foam cells, to either formulation (with or without PS) can increase ApoA-I mediated cholesterol efflux from the cells and possibly reverse the development of foam cells. PS and ω-3 PUFAs have both been implicated as regulators of macrophage cholesterol homeostasis in other *in vitro* studies [17, 25] suggesting that these may be responsible for the changes observed in our study, however, no differences in cholesterol efflux were observed between the formulations with or without PS in our model.

It is now emerging that once in the arterial intima, macrophages can polarise into either pro-inflammatory M1 or anti-inflammatory M2 subgroups and an the accumulation of M1 macrophages may have a detrimental effect on fibrotic plaque stability [8]. In this study we clearly show that either formulation can hinder Raw264.7 macrophage polarisation towards the M1 phenotype as depicted by a reduction in the expression of iNOS and Arg2; two robust markers of the M1 phenotype [34]. This suggests that the formulation may have the potential to help stabilise the fibrotic cap in established atherosclerosis and supports earlier studies that have shown strong links between ω-3 PUFAs and reduced M1 phenotype formation [51, 52].

In conclusion, this is the first *in vitro* study to examine the effect of this combination of nutritional ingredients and provides preliminary evidence to suggest a potential anti-atherosclerotic effect during numerous processes associated with the disease. Furthermore, this study provides support for future *in vivo* animal and/or human studies to elucidate its ability to prevent atherosclerosis.

## Supporting Information

S1 Fig Physiologically relevant doses of the formulation can inhibit IFN-γ induced expression of MCP-1 and ICAM-1 in murine macrophages. (DOCX)

S1 Data Excel spreadsheet of experimental data. (XLSX)

S1 Table Active ingredient inclusion levels of the supplement. (DOCX)

S2 Table Oligonucleotide sequences. (DOCX)
