# Supplementary material for: A Unique Combination of Nutritionally Active Ingredients Can Prevent Several Key Processes Associated with Atherosclerosis In Vitro
Source: PLoS One. 2016 Mar 7;11(3):e0151057. doi: 10.1371/journal.pone.0151057 (PMC4780775; doi:10.1371/journal.pone.0151057)
Supplement: S1 Fig — (DOCX) [file pone.0151057.s001.docx]

**S1 Fig. Physiologically relevant doses of the formulation can inhibit IFN-γ induced expression of MCP-1 and ICAM-1 in murine macrophages.**

******

*****

******

*******

*****

*****

**a**

**b**

******

**VC** **IFN-γ 0.25x 0.5x 1x 2x 4x**

**VC** **IFN-γ 0.25x 0.5x 1x 2x 4x**

Gene transcript levels of MCP-1 (a) and ICAM-1 (b) were assessed in Raw264.7 macrophages that were treated with either vehicle control (VC) or with IFN-γ (250 U/ml) or with IFN-γ (250 U/ml) in the presence of various doses of the complete formulation for 3 hours. Gene transcript levels were calculated using the comparative Ct method and normalized to β-actin levels with values from vehicle treated cells given an arbitrary value of 1. The data are presented as the mean±SEM from three independent experiments. Statistical analysis was performed using a one-way ANOVA with Dunnett T3 post-hoc analysis on log-transformed data where * *p* <0.05, ** *p* <0.01 and *** *p* <0.001.
