# Supplementary material for: A Unique Combination of Nutritionally Active Ingredients Can Prevent Several Key Processes Associated with Atherosclerosis In Vitro
Source: PLoS One. 2016 Mar 7;11(3):e0151057. doi: 10.1371/journal.pone.0151057 (PMC4780775; doi:10.1371/journal.pone.0151057)
Supplement: S1 Table — (DOCX) [file pone.0151057.s003.docx]

S1 Table: Active ingredient inclusion levels of the supplement.

| **Raw Ingredient** | **Active Component** | **mg per dose (of active)** |
| --- | --- | --- |
| Marine Fish oil | $\omega$-3 PUFA | 1100 |
| Cocoa Extract | Flavanols (as catechins) | 200 |
| Mixed Phytosterol Esters | Total Phytosterols | 1300 |
| Water | N/A | N/A |
| Emulsifiers & Flavours | N/A | N/A |
