# Supplementary material for: A Unique Combination of Nutritionally Active Ingredients Can Prevent Several Key Processes Associated with Atherosclerosis In Vitro
Source: PLoS One. 2016 Mar 7;11(3):e0151057. doi: 10.1371/journal.pone.0151057 (PMC4780775; doi:10.1371/journal.pone.0151057)
Supplement: S2 Table — (DOCX) [file pone.0151057.s004.docx]

S2 Table: Oligonucleotide sequences

|  | **Gene** | **Forward Primer (5’ to 3’)** | **Reverse Primer (5’ to 3’)** | **Ref** |
| --- | --- | --- | --- | --- |
| THP-1 and HMDM | GAPDH | CTTTTGCGTCGCCAGCCGAG | GCCCAATACGACCAAATCCGTTGACT |  |
|  | MCP-1 | CGCTCAGCCAGATGCAATCAATG | ATGGTCTTGAAGATCACAGCTTCTTTGG |  |
|  | ICAM-1 | GACCAGAGGTTGAACCCCAC | GCGCCGGAAAGCTGTAGAT |  |
| Raw264.7 | β-actin | ACACCCGCCACCAGTTCGCCAT | CACACCCTGGTGCCTAGGGCGGCCCACGATG |  |
|  | MCP-1 | GCTCAGCCAGATGCAGTTAACG | GCTTGGTGACAAAAACTACAGCTTC |  |
|  | ICAM-1 | ACGTGCTGTATGGTCCTCGG | GTCCAGTTATTTTGAGAGTGGTACAGTA |  |
|  | Arg2 | ATATGGTCCAGCTGCCATTCGAGA | CCACTTCAGCCAGTTCCTGGT | [1] |
|  | iNOS | CAGTTCTGCGCCTTTGCTCAT | GGTGGTGCGGCTGGACTTT | [2] |

**References**

1. Jin Y, Liu Y, Nelin LD. Extracellular signal-regulated kinase mediates expression of arginase II but not inducible nitric-oxide synthase in lipopolysaccharide-stimulated macrophages. J Biol Chem. 2015;290(4):2099-111. doi: 10.1074/jbc.M114.599985. PubMed PMID: 25451938; PubMed Central PMCID: PMCPMC4303663.

2. Chang CS, Sun HL, Lii CK, Chen HW, Chen PY, Liu KL. Gamma-linolenic acid inhibits inflammatory responses by regulating NF-kappaB and AP-1 activation in lipopolysaccharide-induced RAW 264.7 macrophages. Inflammation. 2010;33(1):46-57. doi: 10.1007/s10753-009-9157-8. PubMed PMID: 19842026.
